# Covid‐19 in Brazil in an era of necropolitics: resistance in the face of disaster

**DOI:** 10.1111/disa.12528

**Published:** 2021-12-07

**Authors:** Renata Cavalcanti Muniz, Fiorella Macchiavello Ferradas, Georgina M. Gomez, Lee J. Pegler

**Affiliations:** ^1^ Researcher at the International Institute of Social Studies, Erasmus University Rotterdam the Netherlands; ^2^ Researcher undertaking a joint degree between the International Institute of Social Studies, Erasmus University Rotterdam, the Netherlands, and the University of Brasilia Brazil; ^3^ Associate Professor at the International Institute of Social Studies, Erasmus University Rotterdam The Netherlands; ^4^ Assistant Professor at the International Institute of Social Studies, Erasmus University Rotterdam the Netherlands

**Keywords:** Brazil, civil society, Covid‐19, disaster, inequality, necropolitics, resistance

## Abstract

The Covid‐19 pandemic has been a massive disaster in Brazil, causing more than 350,000 deaths as of April 2021. Moreover, President Jair Bolsonaro suggested that already marginalised groups should take what came to them, as if they were an expendable surplus in his necropolitical perspective. However, civil society initiatives are emerging to tackle the impacts of this crisis. This paper adds to current literature on the forms and levels of resistance to disasters, using primary and secondary data pertaining to three key Brazilian groups: domestic workers; the urban poor in favelas; and indigenous Amazonians. The analysis indicates that their historical, political resistance has been a foundation upon which to develop disaster mitigation and their actions have built on and gone beyond previous modes of organising. More specifically, their responses have replaced a ‘present–absent’ federal government, entailed local, innovative adaptations, led to new public–private sector relations, and may offer the prospect of consolidation.

## Introduction

Governments are expected to respond immediately to disasters to reduce the loss of life. During the Covid‐19 pandemic of 2020–21, authorities worldwide aimed to restrict social contact while strengthening healthcare systems, as recommended by the World Health Organization (2020). Far from adhering to these risk management guidelines, Brazilian President Jair Bolsonaro repudiated them and adopted a discourse that undermined any efforts to mitigate the health hazard. It made Brazil one of the epicentres of the crisis with respect to infections and deaths, with healthcare systems collapsing in several major cities.

To make matters worse, the pandemic arrived at a time of economic failure and political turmoil. In contrast to the government's reaction, groups of poor and marginalised people gradually organised local responses to reduce the risks and protect the most vulnerable. The combination of the pandemic and subsequent ‘non‐governance’ (Ortega and Orsini, 2020) of the disaster changed civil society in unexpected ways. Grassroots organisations all over the country engaged in disaster risk management activities, using scarce resources, and strived to learn new skills and build on previous experiences (Resende, 2021).

This paper aims to comprehend how the most vulnerable social groups have gone from focusing on their original ‘repertoires of contention’ (Tilly, 2010) to become key disaster management actors. It is hoped that it adds a building block to our understanding of the conditions under which resistance evolves in times of necropolitics and how vulnerable groups organise to face disasters. Both trends may combine to promote social justice, even in such a fragile scenario. Our case studies centre on actions taken by vulnerable groups and their mobilisation of governmental and non‐governmental actors within their networks.

First we argue that Bolsonaro deliberately aggravated the disaster to advance his political programme. He heightened his existing harsh, chauvinistic discourse towards black Brazilians, women, indigenous peoples, and Afro‐Brazilian residents of *quilombo* settlements, depicting them as groups that ‘deserved their fate’ in the pandemic. Historically, these groups have been marginalised in Brazilian society and thus were more vulnerable to Covid‐19 (dos Santos et al., 2020). We claim that Bolsonaro mobilised the notion of death to discipline and enhance a system of domination over them, a narrative termed ‘necropolitics’ by postcolonial Cameroonian philosopher Achille Mbembe. The concept refers to the constitution of ‘death worlds’ within the world of the living as a way of subjugating and excluding marginalised segments of the population, as if they were disposable or simply not there (Mbembe, 2003, 2019). It was a key trigger for grassroots organisations to reframe political postures as responses that protected life amidst the disaster.

We explore the relationship between disaster, necropolitics, and political resistance in groups' reactions by focusing on three of the most vulnerable groups in Brazil: domestic workers; the urban poor; and indigenous Amazonians. Domestic workers provide services in family houses on a continuous basis, even if only for a few hours a week; the urban poor are defined as residents of neighbourhoods classified as *favelas* (slums present in most large cities in Brazil); and indigenous Amazonians represent different indigenous groups living in the Amazon region, mainly in the states of Amazonas and Pará. We collected and analysed news articles, official documents, podcasts, and videos. Building on this background material and previous knowledge of historical inequalities in Brazil, our qualitative analysis was based on eight one hour, in‐depth, semi‐structured, online, recorded interviews^2^ with representatives of these groups, conducted between June and July 2020. Interviewees were selected using a snowballing process. We identified key leaders in the groups and then asked them to suggest other names. What these three groups have in common is their existing vulnerability and long‐standing experience of political resistance to pervasive inequalities in Brazil and advocacy for change. These groups were also the target of Bolsonaro's necropolitics.

The second section of the paper elaborates the concepts of disaster, political resistance, and necropolitics, which are illustrated in diagrammatical form. The third section provides essential background, describing the historically constructed inequalities that frame the organisation of collective action among these social groups and how they were affected by the pandemic. The fourth section overlays the actual practice of necropolitics on to this context. The fifth section analyses these groups' local‐level responses to mitigate the risk and prevent further loss of life, that is, the forms of micro‐political resistance to necropolitics and how these may be reshaping these groups and their actions. The paper concludes with some reflections on the impacts of this multi‐causal disaster, cautiously but optimistically assessing the prospects of these modes of resistance engendering improvements in social justice.

## Disaster in times of necropolitics

A disaster can be defined by numbers or by magnitude: when at least ‘10 or more people are reported killed; 100 or more people are reported affected, injured and/or homeless; the government declares a state of emergency; or the government requests international assistance’ (Strömberg, 2007, p. 201). In Brazil, the Covid‐19 pandemic caused more than 50,000 reported deaths (as of April 2021) and the collapse of healthcare systems in most of the country's main cities. While these figures indicate the enormity of the disaster, the national government neither declared a state of emergency nor asked for international assistance, adopting a position of abject denial (Ortega and Orsini, 2020).

In this regard, the impact of disasters depends on historically constructed social conditions (Hilhorst, Boersma, and Raju, 2020) and differentiated vulnerability conditions. Vulnerability is understood here as the propensity to be in a state of injury, including the outcome of sociocultural factors such as ‘unequal power relations’ (Pereira Covarrubias and Raju, 2020, p. 220). Hilhorst, Boersma, and Raju (2020, p. 214) claim that ‘a disaster is the outcome of hazards that are created through human‐nature interactions encountering socially produced vulnerabilities’, or, as expressed by Kelman (2020, p. 15), a disaster is ‘manufactured and implemented by people and their choices’. Hence, we advocate a political approach to analyse the Covid‐19 disaster, one in which the government was both a main factor in aggravating it and a catalyst for political action among civil society. We also follow Pelling and Dill (2006) who argue that ‘a political reading of disaster requires the situating of political action within the wider national and global socio‐cultural and historical contexts in which they occur’. According to the authors, disasters may generate circumstances for potential change in which a ‘discontented civil society’ is a protagonist.

In Brazil's case, the political context is defined by a discourse of denial; the Bolsonaro regime uses the notion of death to undermine marginalised groups and accumulate power. As noted, the narrative reflects Mbembe's (2019, p. 92) concept of necropolitics, defined as ‘contemporary forms of subjugating life to the power of death’. In practice, necropolitics characterises the various ways in which death worlds emerge in our contemporary world and crystalise as forms of existence that resemble a status of the living dead (Mbembe, 2003). ‘The ultimate expression of sovereignty is related to the capacity of deciding who may live and who may die’ (Mbembe, 2003, p. 11). The theory was mobilised to analyse racism and sexism (Wright, 2011; dos Santos et al., 2020), including fascism and Nazism (Mbembe, 2019). Yet, democracies, in general, are seen to internalise these dynamics, becoming ‘societies of separation’:

*Perhaps democracies have always been communities of fellow beings, and therefore […] societies of separation. They may well have always had slaves, a set of people who, in one way or another, are regarded as pertaining to the foreigner, members of a surplus population, undesirables of whom one hopes to be rid, and who, in this way, must be left ‘completely or partially without rights‘* (Mbembe, 2019, p. 42).

The pandemic appealed to Bolsonaro's political programme of necropolitics, providing an optimal opportunity to reaffirm the subjugation of specific groups of people that deserved to be controlled and kept in precarious living conditions (de Moraes Almeida, 2020). Historically, however, marginalised groups in Brazil organised themselves to improve their circumstances, resisting narratives of exclusion. This dynamic thus necessitates that we define more fully the resistance process, as is done below.

Resistance involves acts of intentional and coordinated organisation to confront and oppose power (Hollander and Einwohner, 2004). They take various forms, such as an open display of opposition, a questioning of the rules of the game (pertaining to political or labour relations, for instance), or the articulation of counter‐narratives (see, for example, Lukes, 2021). Resistance is a necessary antecedent to collective action. Scott (1976) and Katz (2004), inter alia, also highlight the operation of ‘everyday forms of resistance’ designed to undermine forms of domination at the local level, but that are not so evident as explicit attempts to resist. But they still seek to weaken a system of domination, questioning its legitimacy and challenging its authority. We argue, too, that resistance extends to developing new capacities and skills to survive and stay safe and expanding networks to strengthen the survival chances of the vulnerable. So, from a political viewpoint of disasters, providing humanitarian aid in a context of necropolitics is an act of resistance: it weakens the government's efforts to exercise power over marginalised groups. Yet, further reactions to these acts may emerge from the regime.

Brazil faced an explosive disaster because of interactions between the Covid‐19 pandemic, necropolitics, and historical social inequalities. The latter led to the organisation of marginalised groups and resistance/collective action against an exclusionary political system. In this setting, marginalised groups further reorganised and transformed themselves into disaster management actors by engaging in risk mitigation initiatives and organising new civil society collectives.

**Figure 1:**
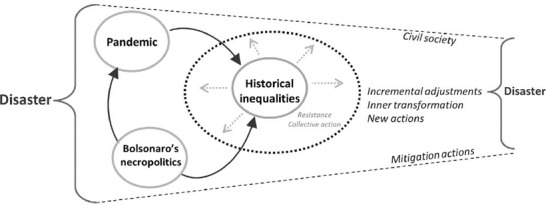
Resistance to survive the Covid‐19 disaster in Brazil, first wave, 2020 **Source**: authors.

Figure 1 illustrates the key elements of our reasoning. Bolsonaro's necropolitics worsened the impacts of the pandemic, reinforcing social disparity. But the more vulnerable were also directly affected by this approach. The combination of these elements enlarged the dimension of the disaster. While resistance and collective action have been part of the history of the country, civil society developed mitigation actions that were fundamental to reducing the impact of the disaster. These new activities led to incremental adjustments in their former course of actions, reorienting their scope and contributing to inner transformations of the organisations involved. In Figure 1, the three groups studied in this paper are represented by civil society.

## Marginalisation and vulnerability: the study context

Brazilian society is characterised by centuries of profound inequalities that affect, to various degrees, most of the population. While the details exceed the scope of this paper, we aim to provide an indication of the situation here. Of the total population, 54 per cent are black (Prudente, 2020), and more than 305 indigenous groups made up a populace of more than 800,000 people in 2010 (the time of the last census). Black and *pardos* ^3^ face the worst living conditions: 44.5 per cent lack access to sewage and only 61.8 per cent finished secondary school in comparison to 76.8 per cent of whites (IBGE, 2019). Black and *pardo* men are most likely to suffer a violent death: 43.4 per cent of homicides in all age groups and 98.5 per cent of homicides of those aged between 15 and 29 years (IBGE, 2019). Blacks and *pardos* also comprise most of the residents of *favelas.*

Covid‐19 arrived in Brazil via the upper socioeconomic classes but quickly spread and brought old and new inequalities to the surface. While people of higher socioeconomic status could afford to be in isolation and work from home, this presented severe challenges to a large part of the population. The first recorded death in the State of Rio de Janeiro, in March 2020, was of a black domestic worker infected after her employers returned from a trip to Europe and tested positive. Tess et al. (2020) pointed to two different pandemics, one spreading rapidly and with high lethality in more impoverished areas of cities such as São Paulo, and another one, slower and less deadly, in wealthier neighbourhoods. Loureiro (2020) analysed deaths in relation to income and showed that the propensity of death due to the virus was higher among residents of vulnerable areas. In São Paulo, 66 per cent of the total number of deaths in the first wave (March–May 2020)^4^ were reported in regions with average salaries below BRL 3,000 (around EUR 500) per month, as compared to 21 per cent in places with an average income of up to BRL 6,500 (about EUR 1,083) per month, and 1 per cent in areas where the average income was more than BRL 19,000 (approximately EUR 3,167) per month (Loureiro, 2020).

It is estimated that Brazil has more than six million domestic workers, of which more than 90 per cent are women and more than 64 per cent are black (Pinheiro et al., 2019). Their labour was regulated by law (as ‘work‘) only on 1 June 2015 (Lei Complementar n° 150), but most of these workers still do not have formal contracts. Monitoring work conditions inside private homes is difficult, leaving these people vulnerable to different types of abuse. Domestic work conditions combined with racist behaviour by employers are underscored by the invisibility of this category of workers, as noted by Cleide Pinto of the Federação Nacional das Trabalhadoras Domésticas (FENATRAD; National Federation of Domestic Workers):^5^

*And then you also have that racist discourse. … Not so long ago, our work was not recognised as work. It took us years of struggle to show that domestic work is work as any other. It is complicated to create consciousness about domestic work being a regular type of work or that there is a person that can be contaminated doing that job.*

In fact, informality and precarious working conditions are a reality for a significant portion of Brazilian society. Of the total number of workers in the country, 41 per cent are informal and without any type of social protection (IBGE, 2020), as highlighted by Manoel Potiguar of the Instituto Peabiru (a non‐governmental organisation (NGO) that empowers civil organisations and seeks to protect biodiversity in the Amazonian State of Pará):^6^

*Belém is the Brazilian capital with the highest number of people working in informal jobs. […] We are talking about fishers that need to sell their daily catch, people that earn their income per day and not per month. All their income depends on their daily work.*

In a domestic work or urban poverty situation, the reality of informal work and the pandemic context presents ambiguities, as noted by Gilson Rodrigues, President of Paraisópolis (the residents' association of São Paulo's second largest *favela*):^7^

*The famous ‘stay home’ does not work for us here. It is not our reality […] quarantine in the* favelas *is the biggest fake news invented. […] The truth is that the worker and resident of the periphery continue to work daily.*

Many informal workers live in *favelas*: communities where state policies are not applied, where there is no proper infrastructure for sewage and rubbish collection, and where sometimes there is also no water. Large families reside in small spaces. Irrespective of the pandemic, these conditions already contribute to the spread of viruses and diseases. The lack of access to clean water, for example, is an old problem in Paraisópolis, a *favela* neighbouring the wealthy area of Morumbi, in the city of São Paulo. In the words of Gilson Rodrigues:^8^

*Several times a day, residents have no water whatsoever. Our organisation had to sue the water provider. They argue that they decrease the water pressure, but in that way, the water does not reach the* favela. *How are we supposed to follow directions to wash our hands frequently if we don't have water to do so?*

Access to clean water is an issue too in the Amazon. Many communities do not have water pipes. Even in urban villages in the region, many communities do not have clean water in their homes. Another problem is the lack of adequate access to healthcare. The Sistema Único de Saúde (SUS; Unified Health System) was created in 1988 under the Constitution of the Federative Republic of Brazil, based on the understanding that it is the state's responsibility to provide healthcare to its population. However, there is a parallel system of private healthcare that is usually used by the upper‐middle class and elites who have private health insurance. SUS, in contrast, is universal and can be accessed by anyone without discrimination or cost. Yet, despite its achievements (such as its local immunisation programme), SUS suffers from a shortage of resources, which compromise its ability to fulfil its goals. Domestic workers, *favela* residents, and the indigenous strongly need SUS for treatment, but care is often difficult to access. Cleide Pinto pointed out that:^9^

*Domestic workers use the public system, stand in a large queue, and most of them die. This is the case not only for the domestic worker but for all poor workers.*

And many indigenous territories can only be reached by boat. Indigenous activist Vandria Borari explained:^10^

*The Amazon region is the size of several countries, and access to the communities is made by boats, which can take many hours or even days. The Brazilian health system is not adapted to access our territories […]. In the region of the Low Tapajós, between two rivers, there are 70 villages and around 500 indigenous territories. In the region of the High Tapajós, there are approximately 8,000 indigenous people. The basic healthcare system is composed of four healthcare professional teams, with one responsible nurse each. To cover this geographical area, the health infrastructure is highly insufficient.*

The complexities of reaching indigenous villages in the Amazon and the historical struggles of the region make indigenous populations even more vulnerable. The federal constitution of 1988 determined that indigenous lands were to be demarcated. Still, the process of demarcation has been insufficient; consequently, indigenous groups are even more vulnerable to physical threats, poor access to services, and necropolitical narratives. For example, many indigenous groups live on land classified as *Extrativist Reserves*, but their goal is to one day have more explicit land rights—that is, have recognised ‘indigenous territory’ where no other occupation is allowed. In addition, the region also suffers disputes over the legality of land occupation related to the role of foreign traders/insurance investors and artisanal miners (Pegler and Widmarck, 2020), as well as volatile border dynamics with neighbouring countries, including problems of drug wars and trafficking (Rapozo, 2020).

As indigenous peoples have had less contact with pathogens than non‐indigenous populations, their situation is aggravated further. Mortality due to Covid‐19 is higher among rural indigenous people than it is among any other group in Brazil. Their Covid‐19 mortality rate is 150 per cent higher than the Brazilian average and 20 per cent higher than that recorded in the country's North Region, with the highest mortality rate overall (Fellows et al., 2020). By January 2021, the number of deaths among indigenous people had reached 936, with 46,834 people from 161 different indigenous groups being infected.^11^ Real numbers are probably higher as cases are underreported. As the guardians and propagators of their history, indigenous elders face the highest infection risks and mortality rates (Dantas, 2020; Mori, 2020).

In terms of the urban impacts of the pandemic in the Amazon, Manaus is one of the worst‐hit cities. After witnessing a dramatic peak in deaths in April 2020, the capital of the State of Amazonas demonstrated the devastating potential of Covid‐19 when its health system collapsed. The municipal administration dug collective graves for bodies as the death rate tripled. Burial services were overwhelmed. In early 2021, this situation became more severe owing to the lack of oxygen available for patients, as Manaus registered a new record‐high in hospitalisation and death rates (G1 AM, 2020).

Domestic workers, *favela* dwellers, and indigenous groups have traditionally been among the most vulnerable in Brazilian society and have been hit most gravely by the pandemic. Furthermore, they have been seriously affected by the dramatic, destructive, and conflictual necropolitics during the Bolsonaro era. The next section underlines the effects of this narrative and process on the study groups. Yet, as the fifth section reveals, they did not accept their fate as a ‘surplus population’, as is suggested by Bolsonaro.

## Overlaying necropolitics on to Covid‐19

While it could be argued that some periods of Brazilian history have experienced necropolitical (actively discriminatory) processes to differing degrees, the term characterises best Bolsonaro's overall way of doing politics, his narrative and his regime. He employed authoritarian rhetoric during the 2018 presidential election campaign, and this has continued throughout his term in office, in government pronouncements and policies. One of his most symbolic gestures is the repeated finger gun motion, which has become his slogan. Not ashamed of praising the dictatorship, Bolsonaro's government has an unprecedented number of military officials (active and non‐active) in ministries and other strategic positions (around 6,000 in 2020)—the most since the end of the Brazilian dictatorship in 1985.

In another demonstration of his exclusionary bias, once elected, Bolsonaro announced that the government was not proceeding with further indigenous territory demarcation (Hirabahasi, 2018), reflecting the priority given to agribusiness. As president, he has approved laws that benefit land‐grabbing and have reduced the resources of government support agencies, such as the Ministry of Health's Secretaria Especial de Saúde Indígena (SESAI; Special Secretariat for Indigenous Health). Within a ‘virtually absent’ or ‘present–absent’ state (Rapozo, 2020), Bolsonaro's *political* stance and narrative increased the precarity of vulnerable groups. We refer to a ‘present–absent’ state because not having a strategy was, in fact, an intentional strategy, a conscious choice that brought indifference towards vulnerable groups to a level of normalcy, exacerbating their invisibility and insecurity. We also agree with Garmany (2009) on the conception of a ‘virtually absent’ state: one which operates undetected but still has a narrative that shapes the minds and behaviours of people (à la Michel Foucault's notion of governmentality), but at the same time, it is not materially present as it does not provide infrastructure and basic services. As Pedro Rapozo underlined:^12^

*Indigenous peoples*, quilombolas, *and the black population, they were always the invisible targets of such necropolitics. The only issue is that these matters are in the spotlight under this government.*

The vulnerable and unwanted became the target of a combination of social control tactics and further impoverishment. Indifference towards their right to be alive was promoted and normalised. Power and control are evident as actions and messages in implicit and explicit respects. Moreover, these policies did not go unnoticed. Affected groups perceive the actions of the current regime as having a clear message:

*We have a government that sends the message that if corona arrives at villages, it should continue there, doing its work, which means to exterminate the indigenous peoples. For the government, this is wonderful as they are anti‐indigenous. They already said that publicly. So, it is clear for us, and we never expect that this government will take any action for us.* ^13^

*We knew there would be many deaths. We knew there would be no ICU or ventilators for everyone, and we knew these deaths would be mainly from slums in Brazil. And that is what the data is showing. The largest number of people dying at this moment in Brazil are black people and from slums.* ^14^

*I watched a report on the TV. They were interviewing an upper‐middle‐class family about the lockdown. But the domestic worker could be seen in the background, working. “Oh, this family is isolated”. But what about that worker back there? Isn't she someone?* ^15^

At the beginning of the pandemic, Bolsonaro disregarded the importance of the virus by declaring that Covid‐19 was ‘just a simple flu’ (Sakamoto, 2020). During the months when the country was witnessing more than 1,000 deaths per day, the president shook hands with supporters, incited people to take ‘preventive measures’ (such as non‐approved drugs), went to gatherings, and showed no empathy for the passing of others. Such behaviour continued despite an increase in the number of deaths.

Bolsonaro's narrative also affected the way in which many citizens perceive the pandemic, as is suggested by Gilson Rodrigues:^16^

*People here think that the virus is for rich people, for those who travelled abroad, or that is just a flu [as mentioned by the president]. People don't understand what it means for them a collapse of the health system, or what working from home is, or what a respirator does. […] The government decided not to explain those things to residents of* favelas. *If the population understood that a collapse in the health system means that if I go to the hospital, I might die at the door, we would have a big problem.*

Reflecting the volatile political climate, the government changed 24 ministers within two years of acquiring its mandate. While these moves may have been used to demonstrate power, they also highlighted a deterioration in the regime's credibility. Other illustrations of power included an attempt to hide the official number of deaths caused by Covid‐19 and the president's imposition of various obstacles to prevent governors and mayors from implementing lockdown measures to contain the spread of the virus.

Subnational and local governments contested the federal government's decisions regarding many measures by following the World Health Organization's recommendations and declaring lockdowns in some cities. Eventually, around June 2020, schools were suspended, public transportation capacity was reduced, and tourist spots and commercial businesses were shut for a time. Some states even closed sub‐state borders and forbade inter‐municipal travel. Others declared a state of emergency. Judicial bodies intervened to try to stop the president from forbidding the states from taking such steps.

Responding to such acts of state autonomy, Bolsonaro decreed that beauty salons, barbershops, and gyms should be put on the list of essential services, forcing them to remain open. He also vetoed a part of a law that gave states responsibility to supply respirators and clean water to indigenous communities (Carvalho, 2020). When corrected by the Supreme Federal Court and forced to allow governors to implement specific measures, the president launched administrative investigations (using the Ministério Público (Public Prosecutor's Office)) of governors of states that openly opposed his line of thought (Jubé, 2020). In the words of Manoel Potiguar:^17^

*The Subnational Government of the State of Pará was one of the first governments to behave against the Federal Government's guidance. The orientation of the president at the beginning was to ignore [the pandemic]. Now the State of Pará is being investigated because it possibly overbilled purchases. […] The investigations of the Federal Government were precisely on the governors who opposed the Federal Government. So, the governors of the State of Pará, Maranhão, São Paulo, and Rio de Janeiro were the ones who suffered the first retaliation by the Federal Police.*

Furthermore, measures initiated at the legislative level were sometimes implemented without considering the particularities of the people in need. For example, an emergency cash support scheme was approved by the National Congress in April 2020. Besides having a value of BRL 600, approximately one‐half of the minimum wage (BRL 1,100), there were also problems with its execution. Notably, registering online to receive support was a challenge for most people, owing to a lack of access to the internet. And withdrawing the money from the bank was burdensome in villages with only one ATM (automated teller machine), such as in Breves in the State of Pará. Manoel Potiguar remarked:^18^

*There is only one agency from the public bank Caixa Econômica [Federal] to assist more than 50,000 people. […] The majority of the population of the city of Breves does not have access to the internet. So, how is it possible to assure access to emergency assistance for these people? […] Breves is a city that acts as a pole. People from all the municipalities of the rest of the Marajó Island go to Breves for cash withdrawals.*

In summary, a disaster struck a country already structured around systemic inequalities, which were accentuated as the pandemic worked its way through Brazil. The situation was further aggravated by a necropolitical narrative, omissions, and the priorities and policies of the Bolsonaro regime. The administration failed to help vulnerable groups and employed strategies that further challenged their survival. Yet, Brazilian civil society has a strong history of organising resistance to an exclusionary system. The next section analyses the possible significance of these responses.

## Reframing and reorganising resistance

The Bolsonaro government opted for a policy of not having an emergency plan to protect the marginalised, consistent with our interpretation of a ‘virtually absent’ state. Its necropolitical approach blamed these groups for living conditions that facilitated the contagion. However, local social organisations representing those groups reacted by taking on typically federal government functions. New networks, often involving members of local state agencies, NGOs, and the private sector, were created while others reframed their missions and actions. A mixed model of action–reaction, building on the past and sewing seeds for potential new social movements, seems to have emerged, as summarised in Table 1 (especially by comparing the ‘Resistance’ column with the ‘Reframed action’ column).

Although similar pressures applied to all of these precarious groups, they are not homogeneous entities, and their vulnerabilities vary. They also differ in their organisational basis and form (see columns two to four in Table 1). For instance, Brazilian domestic workers are organised in unions, which are part of the country's official legal framework, one that is formally equipped to fight for better conditions. In contrast, in *favelas*, organisation is an invisible form of governance, ‘an amalgam of household and neighbourhood‐level activities and networks’ (Hilhorst, Boersma, and Raju, 2020, p. 214) that seek better livelihoods and urban infrastructure. With a robust territorial component, governance in the *favelas* depends on unrecognised non‐state structures and alliances with other groups (Zibechi, 2012). In a similar vein, territorial rootedness is also essential among indigenous collectives (Zibechi, 2012). Still, the concept of territory has a more profound historical and symbolic value (Prezia, Maestri, and Galante, 2019) since it involves resistance to challenges and the preservation of culture. Moreover, there are actually numerous indigenous associations, which are fragmented and dispersed as they emerged in diverse regions of Brazil in the 1980s; they became even more plentiful after being registered as legal entities, such as Povos Indígenas no Brasil.^19^

FENATRAD played a crucial role in fighting for domestic workers by informing them of their rights and organising seminars and conferences. FENATRAD, which was created in 1936 and encompasses 22 workers' unions in 13 states, estimates that at least 1.5 million domestic workers lost their jobs during the Covid‐19 crisis. To resist the pandemic, it built on traditional union activities and, in the absence of support from the federal government, organised online events and national campaigns and employed informal means, such as contacting workers via WhatsApp messages. Cleide Pinto said:^20^

*I took the files of the domestic workers that were at the union and started to send them WhatsApp messages. Then they started talking about the difficulties they were going through. The person who has a car takes the food baskets for the others […] these are solidarity bonds from one to the other.*

An example of their political resistance was the campaign entitled ‘cuida de quem te cuida’ (‘care for those who take care of you‘). As part of this drive, the union pressured politicians to prevent the classification of domestic work as essential, which would give employers the right to fire those who did not want to work during the pandemic. Yet, despite the participation of some famous voices, the campaign did not prevent the governors of some states (Ceará, Pará, Maranhão, and Rio Grande do Sul) from declaring domestic work as essential. Even in states where the job was not officially considered to be so, most domestic workers continued to work because of fear of being fired.^21^ Ironically, a category of workers who had to fight to have their job recognised as formal work in the past were now deemed essential. The campaign also collected personal protective equipment (PPE) and delivered it to workers. People such as Cleide Pinto of FENATRAD coordinated a national campaign to distribute essential goods to domestic workers who had lost their jobs.

Many domestic workers face a double burden of poor working conditions and living in informal urban settlements. In the *favelas*, the response to the impacts of this situation came from different fronts. Before the pandemic, Gilson Rodrigues of Paraisópolis was president of the local community centre and coordinator of the ‘G10 das Favelas’ network, which formed to support new business ideas emerging in different *favelas* throughout the country. The network reorganised its activities during the pandemic to support and empower residents more actively through the concept of ‘street presidents’. Volunteers were allocated a street in the neighbourhood to distribute food and first aid products to residents. At a peak in 2020, G10 das Favelas had 658 volunteers serving as street presidents. Rodrigues became known as the ‘Prefeito’ (‘Mayor‘) of Paraisópolis (Caseff, 2020), and the network idea spread to other *favelas* in Brazil.

The pandemic also led groups in the *favelas* to start engaging in some new activities, compensating for what the state was not doing. For example, they organised a crowdfunding campaign to hire private ambulances to transport *favela* residents, as the national public ambulance system does not reach them. Gilson Rodrigues said:^22^

*SAMU [Serviço de Atendimento Móvel de Urgência; Mobile Emergency Medical Service] does not get to Paraisópolis. It did not do so even before the pandemic, even less now. It costs us BRL 5,000 a day. There are three ambulances, two doctors, two nurses, and two rescuers. Around March 23^rd^ [2020], these ambulances had received already 3,753 calls. So, it is imperative to have this service.*

In terms of learning and gaining skills, G10 das Favelas trained 240 first aid brigades in communities and organised information campaigns and hygiene procedures to help prevent the transmission of Covid‐19. Two schools in the neighbourhood of Paraisópolis were transformed into centres for hosting those who tested positive, allowing them to be isolated, with food, a television room, and some personal space. In this way, *favela* communities shifted from promoting local empowerment and entrepreneurship to providing humanitarian aid, emergency accommodation, and healthcare training.

As mentioned, domestic workers are usually residents of the *favelas.* To support the number facing unemployment, G10 das Favelas also set up the ‘adote uma diarista’ (‘adopt a domestic worker‘) programme, which provided financial resources, hygiene materials, and food to more than 1,000 informal cleaners in Paraisópolis. This initiative was such a success that it was replicated in 14 states. Yet, with the passing of the months, resources dried up and government support never arrived, so the programme lost impetus.

The accumulation of new capacities to provide humanitarian aid also occurred among indigenous people in the Amazon. Vandria Borari said of the Indigenous Council Tapajós‐Arapiuns (CITA):^23^

*CITA is assuming the role of the Brazilian state, building solidarity campaign networks between non‐governmental organisations, universities […] mobilising the government's own institutions […]. This articulation is coming from the indigenous themselves, not from the government.*

These groups are well connected and could move existing networks in new directions, such as to acquire additional resources.^24^ With an inoperable federal state, their focus moved to the subnational and municipal levels of government, which adopted a more prominent, direct role in supporting these groups. This involved partnerships among civil society, indigenous leaders, and private and local public sector agencies.

For example, CITA, initially created to promote public policies on education and health among indigenous peoples, gained experience in using crowdfunding tools for food distribution and the donation of first aid products. Anderson Tapuia of CITA commented:^25^

*Since the first case, with the death of our warrior Borari in Alter do Chão, we felt helpless and did not know what to do. From that came the idea to pressure public health agencies at the local, state and federal level, but we know that processes are very slow. […] So, we decided to mobilise ourselves also at the local and regional levels. Different indigenous groups started working from their organisations, ensuring that the public policies would work.*

The role of indigenous group organisations was also reoriented to include controlling the entrance of non‐indigenous persons to indigenous lands. Using an indigenous academic directory, they monitored, with the help of the Federal University of Western Pará, indigenous students in urban areas and offered hygiene kits, food, and financial help as a strategy to prevent them from going to their villages and possibly acting as spreaders of the virus.

Besides, indigenous groups arranged ambulance boats through partnerships with local organisations. For instance, the Projeto Saúde e Alegria ('Health and Happiness Project') is a civil society endeavour that brought (since 1987) healthcare to isolated communities in the Amazon. During the pandemic, resources were steered towards the Com Saúde e Alegria Sem Corona ('Health and Happiness without Corona') campaign, supporting the public system in reaching communities living next to rivers. In partnership with other local civic organisations, they also manufactured and distributed masks. And, in addition to their traditional assistance and advocacy roles, they have engaged in disaster mitigation activities owing to the absence of the government.

In terms of new activities and skills, all three groups—domestic workers, *favela* communities, and indigenous peoples—became more active on social media, organising online public events to denounce their situation. They often invited artists to support their cause. Personal leadership, collective action, and community care were vital in establishing informal networks. Efforts were facilitated by the use of digital tools, such as WhatsApp and crowdfunding websites.

Where possible, groups used their new skills to highlight the importance of their profession. In the more informal setting of urban poor and distanced indigenous communities, evolving strategies built more on their cultural and networking bonds. Among indigenous groups, online platforms were created through a partnership between the Articulação dos Povos Indígenas do Brasil (Articulation of Indigenous Peoples of Brazil) and seven other indigenous organisations and socio‐environmental institutes, with the support of alternative media (Emergencia Indigena).^26^ To denounce rights violations, the same network organised the Relatório de Violações aos Povos Indígenas no contexto da pandemia do Covid‐19 no Brasil (‘Report of Violations against Indigenous Peoples in the Context of the Covid‐19 Pandemic in Brazil‘).

**Table 1:** Summary of resistance actions by civil society

Organisation	Original nature of collective	Governance	Claim	Resistance: collective action before Covid‐19	Reframed action, during Covid‐19 outbreak	Shifts in the level of engagement, before and after Covid‐19
Domestic workers	Labour union	Formal	Working conditions	Lobbying for better rights Information on workers' rights Subnational level	Active contact with individual workers in need Lobbying against the ‘essential’ character of domestic work during the pandemic Creation of protection network for workers Distribution of PPE New partnerships with civil society Information campaigns Crowdfunding campaigns at the national level	Subnational → local and national
Urban *favelas*	Neighbourhood organisation	Less formal non‐state authority	Territorial embeddedness	Lobbying for infrastructure and basic services Network of favelas to support business (G‐10) Empowerment programmes, training Lobbying – local level	Community mobilisation—planning, organisation, training, and coordination of street presidents (600+) Hiring ambulances and medical staff Training 240 brigades Creation of centres for isolation—redefinition of closed spaces Sharing experiences National repercussion—actions replicated in 14 states and reports in international media Reinforcement of national networks New partnerships with the private sector and civil society Information campaigns Crowdfunding campaigns, supporting the unemployed	Local → National
Amazon communities	Indigenous association	Fragmented and dispersed legal entities	Territorial rootedness, culture, and ethnicity	Lobbying for land rights, recognition of basic social and healthcare rights, and provision of basic services National level, but involving many diverse groups	Movement control in and out of villages and indigenous territories Support for students living in cities Food purchases, logistics, and distribution Ambulance boats Manufacturing and distribution of masks Monitoring, management, and dissemination of Covid‐19 statistics Development of online platforms Creation of channels for reporting violations during the pandemic and for preserving oral‐based memories New partnerships with the private and public sectors and civil society Information campaigns—translation of informative material into indigenous languages Crowdfunding	National → Subnational & Local

**Source**: authors.

The dissemination of information was one of the new areas of action that became central in endeavours to resist the governments' efforts to reduce access to information, especially the number of deaths. Covid‐19 cases are confirmed through direct contact with local indigenous leaders and associations, including indigenous people living in urban areas, who are not counted by the SESAI. The ‘Covid‐19 e os Povos Indígenas’ digital platform was created to monitor the pandemic.^27^ It is available in more than 30 languages, updated daily, and provides geo‐referenced data on isolated indigenous peoples and a vulnerability index to prioritise assistance to communities in critical conditions. In turn, the Comitê Nacional pela Vida e Memória dos Povos Indígenas (National Committee for Indigenous Life and Memory) was established to preserve the knowledge of indigenous peoples; something usually passed down orally through the generations.

The government's attempt to conceal information also led to a combined and unprecedented reaction by national media, newspapers, and television channels. A new media network (G1 et al., 2020) was set up in June 2020 to monitor the number of deaths. It worked collaboratively in gathering information in the 26 states and the Federal District, so that Brazilians could monitor the evolution of Covid‐19.

In sum, domestic workers, communities in the *favelas*, and indigenous people reframed their existing collective organisations to protect life and to resist necropolitics. They accumulated new skills and capacities for action that may translate into meaningful self‐transformation. Regardless of whether organisational capacities were part of formal or informal structures or linked to labour rights protection, daily support in the neighbourhoods, or the demands for indigenous land, cultural preservation, and assistance services, they all reinvented themselves (see the penultimate column in Table 1) to mitigate the effects of the disaster (one compounded by existing inequalities), a political–economic crisis, and the heavy hand of a necropolitical regime. In many instances, their actions also led to escalation up to the sectoral or regional level (see the last column in Table 1). To survive, civil society groups engaged in unprecedented cooperation with other actors, increased their autonomy, and became more self‐reliant.

## Conclusion

Our case studies illustrate the type of actions taken by the most vulnerable groups during the Covid‐19 crisis and how they mobilised both governmental and non‐governmental actors within their (further evolving) networks. Some of them fulfilled functions and services typically provided by the state, and by adopting these outside the realm of the state, civil society groups implicitly weakened the system of domination, questioned its legitimacy, and undermined its authority. In the context of the pandemic and necropolitics, we argue that resistance extends to developing new capacities and skills to survive and stay safe and the expansion of networks to strengthen the position of the vulnerable. In many cases, the adaptation process saw them reaching out to new scales, achieving different types of influence, and pursuing new courses of action. Organised groups of domestic workers, *favela* communities, and indigenous Amazonians performed many of the tasks of the federal government. In the process, they seemed to increase their legitimacy and strengthen their alliances and political power.

Overall, historical inequalities set the stage for the severity of the Covid‐19 disaster in Brazil in two important ways. On the one hand, they were the basis of collective action by marginalised groups, resisting a long tradition of neglect and exclusion by the state. They lived in a ‘virtually absent, present–absent’ state, one that denied them access to public services and infrastructure while subjecting them to notions of subjugation as normalcy. Their ‘normal’ place was on the margins of Brazilian modern statehood and society. The necropolitical regime of President Bolsonaro underplayed the risks of the pandemic yet incorporated the Covid‐19 hazard as part of a disciplinary discourse in which the marginalised deserve what comes to them, including death. The disaster thus became an additional force of subjugation, as well as of renewed necessity to resist.

On the other hand, dialectically, the regime's necropolitics were a trigger for new adaptations to these existing resistance structures. The most vulnerable social groups grew from their original ‘repertoires of contention’ (Tilly, 2010) into disaster management actors. They made this possible by relying on their existing networks of support, which they redeployed in this time of crisis. Their actions simultaneously promoted the protection of life and their demands for social justice. They claimed a place in society that would not be subjugated by necropolitics or located on the fringes of society. In short, in a time of necropolitics and pandemic, the protection of life becomes part of their repertoire of contention.

Whether these initiatives will have long‐term outcomes that help to counterbalance this authoritarian regime is not yet clear. Historically constructed hierarchies and inequalities in Brazil underline both why these local innovations have emerged and their limits as continuing responses. Further necropolitical aggression against these groups may also occur. Then again, other forms of institutional action to try to place limits on the excesses of the regime may also emerge.

This research suggests that these responses have replaced a ‘present–absent’ federal regime that adopted a counterproductive strategy that aggravated the disaster. The innovative local adaptations of organised groups led to new public–private sector relations, offering the prospect of their dispersal and consolidation. These observations may also provide valuable clues in understanding political resistance as an adequate basis for humanitarian aid during disasters. Such emergency assistance is locally relevant, self‐organised, and reaches the proper beneficiaries, who become actors and not victims. But it remains to be seen whether such endeavours lead to new types of collective action and alternative civil organisation for social justice in Brazil.

## Acknowledgements

This work was supported by the International Institute of Social Studies, Erasmus University Rotterdam (grant number: RIF‐5/18202010.037) and by the Netherlands Organisation for Scientific Research (grant number: 453‐14‐013).

## Data availability statement

The data that support the findings of this study are available from the corresponding author upon reasonable request.
